# Acidosis slows electrical conduction through the atrio-ventricular node

**DOI:** 10.3389/fphys.2014.00233

**Published:** 2014-06-25

**Authors:** Ashley M. Nisbet, Francis L. Burton, Nicola L. Walker, Margaret A. Craig, Hongwei Cheng, Jules C. Hancox, Clive H. Orchard, Godfrey L. Smith

**Affiliations:** ^1^British Heart Foundation Glasgow Cardiovascular Research Centre, Institute of Cardiovascular and Medical Sciences, University of GlasgowGlasgow, UK; ^2^School of Physiology and Pharmacology, Medical Sciences Building, University of BristolBristol, UK

**Keywords:** atrio-ventricular node, optical mapping, acidosis, right atrium, atrio-ventricular block, bradycardia

## Abstract

Acidosis affects the mechanical and electrical activity of mammalian hearts but comparatively little is known about its effects on the function of the atrio-ventricular node (AVN). In this study, the electrical activity of the epicardial surface of the left ventricle of isolated Langendorff-perfused rabbit hearts was examined using optical methods. Perfusion with hypercapnic Tyrode's solution (20% CO_2_, pH 6.7) increased the time of earliest activation (T_act_) from 100.5 ± 7.9 to 166.1 ± 7.2 ms (*n* = 8) at a pacing cycle length (PCL) of 300 ms (37°C). T_act_ increased at shorter PCL, and the hypercapnic solution prolonged T_act_ further: at 150 ms PCL, T_act_ was prolonged from 131.0 ± 5.2 to 174.9 ± 16.3 ms. 2:1 AVN block was common at shorter cycle lengths. Atrial and ventricular conduction times were not significantly affected by the hypercapnic solution suggesting that the increased delay originated in the AVN. Isolated right atrial preparations were superfused with Tyrode's solutions at pH 7.4 (control), 6.8 and 6.3. Low pH prolonged the atrial-Hisian (AH) interval, the AVN effective and functional refractory periods and Wenckebach cycle length significantly. Complete AVN block occurred in 6 out of 9 preparations. Optical imaging of conduction at the AV junction revealed increased conduction delay in the region of the AVN, with less marked effects in atrial and ventricular tissue. Thus acidosis can dramatically prolong the AVN delay, and in combination with short cycle lengths, this can cause partial or complete AVN block and is therefore implicated in the development of brady-arrhythmias in conditions of local or systemic acidosis.

## Introduction

Relatively slow electrical conduction through the AVN normally ensures correct timing of atrial and ventricular contraction (Meijler and Janse, 1988). The AVN also plays a protective role in some abnormal cardiac rhythms while contributing to the generation of others. For example, during atrial fibrillation, one of the most common arrhythmias in humans, the AVN protects the heart by limiting the frequency of impulse transmission to the ventricles. In contrast, during some supraventricular tachycardias, the AVN is involved as part of the conduction circuit sustaining the arrhythmia (Sheinman et al., 1982). The critical roles of the AVN in normal and aberrant electrical conduction make it a region of major clinical importance.

Pathological conditions such as myocardial ischemia and systemic acidosis result in exposure of the heart to acidic conditions (Elliott et al., 1992). Lowered pH can result in detrimental consequences that include a reduction in cardiac contractility and development of cellular triggers for arrhythmias [early and delayed after-depolarizations (EADs and DADs)] and predispose toward re-entry (Orchard and Cingolani, 1994). The effects of acidosis on ventricular tissue and cells have been investigated extensively (Orchard and Cingolani, 1994; Hulme and Orchard, 2000; Crampin et al., 2006). In contrast, the electrophysiological consequences of acidosis for other cardiac regions are less well understood. The information that is available suggests that effects of acidosis on the cardiac conduction system may be important. For example, Purkinje fiber excitability is altered by exposure to low pH (Brown et al., 1978), whilst the spontaneous rate of sinoatrial nodal cell and small tissue preparations shows marked pH sensitivity (Satoh and Hashimoto, 1983; Satoh and Seyama, 1986). Work on the intact rat heart implicates the AVN as a major cardiac site of action of acidosis (Aberra et al., 2001); electrocardiogram measurements have shown both heart rate reduction and propranolol- and atropine-insensitive prolongation of the P-R interval with acidosis (Aberra et al., 2001), unaccompanied by significant changes to the QRS complex. Further evidence for sensitivity of the AVN to acidosis comes from the observation that experimental hypoxaemic/hypercapnic hyperventilation and apnoea produced in cats by inhalation of increased CO_2_ is accompanied by acidosis and high-level AV conduction block (Tomori et al., 1997). Furthermore, non-compensated lactic acidosis associated with salicylic acid (aspirin) poisoning has been associated with third degree AV block, whilst toxic adenoma of the thyroid gland has been associated, albeit rarely, with complete heart block accompanied by metabolic acidosis (Pena-Alonso et al., 2003).

In a prior study, we have demonstrated both a direct, negative chronotropic effect of acidosis on spontaneously active cells isolated from the rabbit AVN and a propensity for low pH to inhibit ionic currents in these cells (Cheng et al., 2009). Although this supports the notion that AVN cell function is inherently sensitive to low pH, there is still little information available at present on direct effects of acidosis on electrical conduction through the AVN. Consequently, the present study was undertaken to address this deficit through experiments performed on rabbit intact hearts and isolated right atrial preparations containing the AV junction, compact AV node and penetrating His bundle.

## Methods

### Whole heart preparation

The investigation conforms to the Guide for the Care and Use of Laboratory Animals published by the US National Institutes of Health (NIH Publication No. 85–23, revised 1996). All procedures involving animals were approved by Glasgow University ethics board in accordance with the Home Office (UK) Code of Practice and Animals (Scientific Procedures) Act 1986 guidelines. Adult male New Zealand White rabbits (weight 3–3.5 kg) were killed by intravenous injection of 0.5 ml/kg Euthatal (sodium pentobarbitone 200 mg/kg, Rhône Mérieux) with 500 IU of heparin. Hearts were then rapidly excised and Langendorff-perfused with Tyrode's solution (see below for composition) at 37°C and at constant rate (40 ml/min) using a Gilson Minipuls 3 peristaltic pump. Perfusion pressure was monitored by a transducer in the aortic cannula. Pairs of platinum hook electrodes were placed in the low right atrium. The LV free wall epicardium was stimulated via a pair of bipolar electrodes, which could be moved to different positions on the epicardial surface (*n* = 5).

### Isolated right atrial preparation

In a separate set of experiments (*n* = 11), an isolated right atrial preparation was prepared by removal of all ventricular tissue, followed by an incision around the crest of the right atrial appendage which, when folded open, exposed the endocardial surface of the right atrium and inter-atrial septum. Remaining left atrial tissue was removed, leaving a section of tissue containing the triangle of Koch, the Crista Terminalis, the right atrial appendage and a small section of right ventricle and inter-ventricular septum. The sinus node was left intact. This preparation was pinned onto a Sylgard plate and superfused with oxygenated Tyrode's solution (40 ml/min, 37°C). A bipolar silver wire stimulating electrode was positioned on the endocardium at the right atrial appendage near the sinus node. The spontaneous sinus cycle length was recorded in every experiment. Thereafter, the atrium was driven using 2 ms voltage pulses with an amplitude of 2× threshold. Two silver wire bipolar extracellular recording electrodes monitored surface electrograms from the low inter-atrial septum and the His bundle region; the inter-electrode distance was fixed at 14 mm. Electrograms were high pass (40 Hz) filtered by the MAP amplifier before digitization.

The rate dependent properties of the AVN were then determined using standard pacing protocols as follows: Atrio-Hisian (AH) interval and Wenckebach cycle length were derived using 16 stimuli at the basic PCL followed by a 1 s pause, with the cycle length decrementing by 5 ms intervals after each pause. The atrial effective refractory period was defined as the longest S1S2 interval that failed to elicit an A2 response, and was determined using a protocol that consisted of 16 beats at the basic PCL followed by one premature beat followed by a 1 s pause. The premature beat coupling interval was decremented by 5 ms steps until atrial refractoriness (defined as the longest S1S2 interval that failed to elicit an A2 response) occurred. The AVN effective refractory period (ERP) is defined as the longest A1A2 interval that failed to elicit an H2 response. The AVN functional refractory period (FRP) is defined as the shortest H1H2 interval achieved by premature stimulation. These protocols allow AVN conduction and refractory curves to be constructed, from which parameters of AVN function can be derived.

### Optical mapping

The heart was placed in a custom-made Perspex chamber (Caldwell et al., 2007; Walker et al., 2007), which allowed control of bathing solution temperature and recording of global ECG via wall-fixed electrodes. The heart was loaded with a bolus injection (200 nmoles injected into the perfusate over 30 s) of the voltage sensitive dye RH237 (Molecular Probes, OR USA) dissolved in DMSO (2 mM).

The anterior surface of the heart was illuminated by 535 ± 25 nm light (interference filter, Comar Instruments Ltd, UK) from four 100 W tungsten-halogen lamps. Light emitted from the heart was collected using a camera lens (Nikon 85 mm, NA 1.4), passed through a 695 nm long-pass filter, and the image focused onto a 16 × 16 photodiode array (C4675–102, Hamamatsu Photonics UK Ltd) at × 1.2 magnification. Each diode had a sensing area of 0.95 × 0.95 mm with a distance from center to center of 1.1 mm, so that each diode detected light from a 0.8 × 0.8 mm area of left epicardium, and the full array recorded from an area 15 × 15 mm. Images were collected at 1 kHz and the digitized data samples were stored on disk and analyzed using software written in IDL (Interactive Data Language, Research Systems Inc). All optical signals were conservatively filtered, with a spatial Gaussian filter with a radius of 2 pixels, in accordance with the principles set out in Mironov et al. (2006).

The sequence of activation through the AV junction in the right atrial preparation at normal pH and during acidosis was also studied using optical mapping. Hearts excised were loaded with RH237 as described above. The isolated right atrial preparation was prepared as described above and mounted in a custom-made chamber to allow superfusion with Tyrode's solution and simultaneous recording of surface electrograms and optically derived action potentials using a Red Shirt NeuroCCD SMQ camera (Redshirt Imaging Inc, GA USA). The image of the AV junction was focused onto the CCD array such that an area of 14.5 × 14.5 mm^2^ at a spatial resolution of 40 × 40 pixels (3 kHz frame rate) was imaged. The AV node signals, recorded with the RedShirt system, were additionally filtered with a temporal running median filter (width = 5 and 3 samples). The preparation was paced using the protocols described above at pH 7.4, 6.8, and 6.3. These values encompass the range reported for the heart during pathological conditions, such as myocardial ischemia (Elliott et al., 1992).

### Solutions

The standard Tyrode's solution used in these experiments comprised (in mmol/L): Na 134.5, Mg 1.0, K 5.0, Ca 1.9, Cl 101.8, SO_4_ 1.0, H_2_PO_4_ 0.7, HCO_3_ 20, acetate 20 and glucose 50, equilibrated with 95% O_2_/5% CO_2_, pH 7.4. Acidic solution was generated by equilibrating the standard Tyrode's solution with 80% O_2_/20% CO_2_, pH 6.7. An alternative Tyrode's-based solution with the following composition (mmol/L): Na 134.5, Mg 1.0, K 5.0, Ca 1.9, Cl 101.8, SO_4_ 1.0, H_2_PO_4_ 0.7, HEPES 20, acetate 20 and glucose 50 was used in experiments involving an open chamber, and was adjusted to 7.4, 6.8, and 6.3 by the addition of HCl. The whole heart experiments were performed using CO_2_/HCO_3_ buffer, in which slowing of conduction across the AVN was evident during acidosis. To study this slowing in more detail we examined AV nodal conduction in isolated tissue experiments in an open chamber. This configuration prevented the use of a volatile pH buffer, so that HEPES was used for these experiments. The effect of acidic media generated by high PCO_2_ on AV conduction in intact hearts was similar in magnitude to that seen when the equivalent pH was generated by HEPES based solutions on isolated AVN. For this reason we concluded that the predominant effect is due to pH rather than PCO_2_. Avoiding CO_2_/HCO_3_ buffers allowed a range of pH values to be used easily *in vitro*. The HEPES based solutions were oxygenated with 100% O_2_. 50 mM glucose was added to both solutions to slow oedema (Choi et al., 2006). For measurements from isolated heart motion artifact was reduced with 3 μmol/L cytochalasin-D (Sigma Aldrich, UK). This has previously been demonstrated to have no significant effect on cardiac activation parameters in rabbits over the range of stimulus frequencies used in this study (Walker et al., 2007). The heart was loaded with 100 μl RH237 (Molecular Probes) dissolved in DMSO (1 mg/mL).

### Data recording and statistical analysis

Baseline data were obtained under control conditions (5% CO2/ pH 7.4). The preparation was then superfused with the test solution and data recorded after 10 min. To determine reversibility of the effects, the solution was returned to control and data recorded after 10 min. Results are expressed as mean ± SE. Comparisons were made using ANOVA, paired or unpaired *t*-tests, as appropriate. A two-tailed *p*-value of less than 0.05 was considered statistically significant.

## Results

### The effect of hypercapnic acidosis on epicardial activation

**Figure 1:**
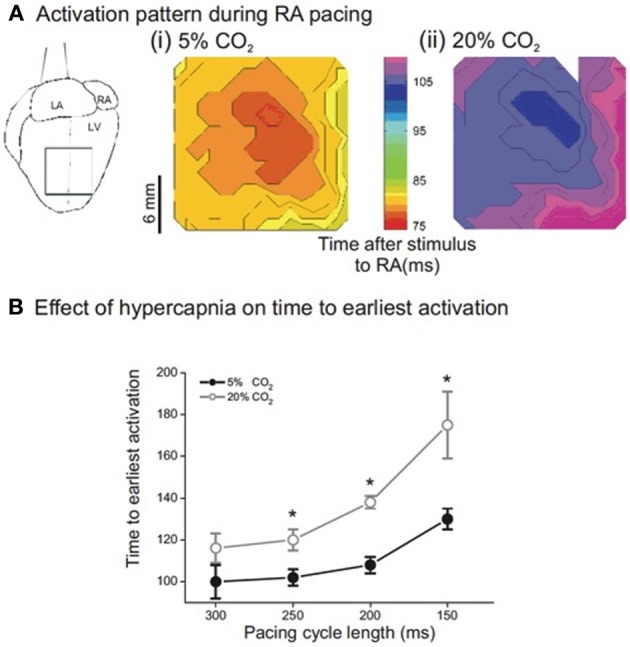
**(A)** Sample isochronal maps of left ventricular (LV) epicardial activation during right atrial pacing. (i) Shows the activation pattern during normal pH conditions. The color bar indicates the time from stimulus to epicardial activation in ms. (ii) Is the same area of LV epicardium during acidic conditions—note that activation is delayed but the pattern is unchanged. **(B)** Effect of pacing cycle length on time to earliest epicardial activation. There was significant slowing of conduction from a PCL of 300 ms down to 150 ms in both normal and acidic conditions. In addition, there was significant slowing in acidic conditions when compared to the same PCL in normal conditions. (*n* = 5 except PCL 300 ms where *n* = 4, ^*^p < 0.02).

Figure 1A shows representative isochronal maps of the epicardial surface of a Langendorff-perfused rabbit heart loaded with RH237, monitored optically during atrial pacing (PCL 300 ms). The left panel shows the pattern of activation at control pH; the right panel during hypercapnic acidosis. Although acidosis had almost no effect on the point of breakthrough or subsequent pattern of activation, the time to breakthrough, and thus the time to activation of the left ventricular (LV) epicardial surface, was clearly prolonged during acidosis. Figure 1B shows that this effect was rate dependent: at control pH, decreasing PCL resulted in a small (though significant) increase in the time to earliest epicardial activation. During acidosis, the time to earliest epicardial activation was prolonged at each PCL, with the effect becoming more pronounced at shorter PCL. Thus, acidosis increased the delay between right atrial stimulation and earliest LV epicardial activation.

To investigate the site of delay in more detail, intra-atrial conduction was measured as the time from activation in the standard right atrial position (low paraseptal) to recording in the high right atrial electrode (0.75 cm diagonally distant toward the right atrial appendage) at each PCL in normal and acidic conditions. The top two rows of Table 1 show that hypercapnic acidosis did not significantly lengthen intra-atrial delay across the range of PCLs studied. The effect of acidosis on conduction through the ventricular wall was monitored by pacing from the ventricular endocardium via a plunge bipolar electrode through the LV free wall and monitoring earliest LV epicardial activation using optical mapping. The lower two rows of Table 1 show that PCL had no significant effect on transmural conduction time at either pH, and that pH did not significantly lengthen trans-mural conduction at any of the PCLs studied. To assess peak longitudinal conduction velocity (CV) on the epicardial surface, a bipolar contact electrode on the LV epicardium was used, and CV was assessed at a 5 mm radius from the activation point. PCL had no significant effect on CV at either pH, and pH had no significant effect on trans-mural conduction at any of the PCL studied (not shown). Taken together, these data provide evidence that the observed acidosis-induced atrial-LV epicardial delay was not due to intra-atrial or intra-ventricular delays, and that the delay therefore represents slowed atrial-ventricular conduction, which could arise due to slowed conduction through the AVN.

**Table 1:** **Comparison of the intra-atrial and transmural conduction time (ms) under normal (5% CO_2_) and hypercapnic (20% CO_2_) conditions in isolated Langendorff-perfused rabbit hearts (*n* = 5) at four pacing cycle lengths (PCL)**.

**PCL**	**300 ms**	**250 ms**	**200 ms**	**150 ms**
Intra-atrial time (5%)	16.6 ± 0.5	17.7 ± 0.5	18.2 ± 0.7	19.6 ± 1.2
Intra-atrial time (20%)	18.3 ± 0.7	19.2 ± 0.7	19.6 ± 0.5	21.0 ± 0.5
Transmural time (5%)	17.0 ± 3.0	21.0 ± 3.8	21.0 ± 3.8	22.3 ± 3.4
Transmural time (20%)	17.0 ± 3.1	24.5 ± 3.3	24.3 ± 3.3	24.8 ± 3.1

**Figure 2:**
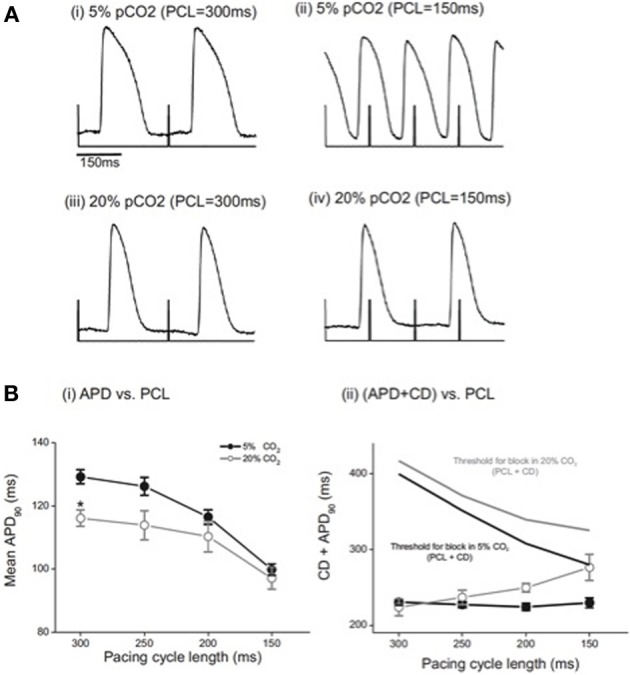
**(A)** Examples of optical action potentials recorded from the epicardial surface under control conditions (5%CO_2_, pH 7.4) and during acidosis (20% CO_2_, pH 6.7). With right atrial pacing, and recording of optical action potentials on the epicardial surface of the ventricle, note the 2:1 relationship of the stimulus artifact to the optical action potential at PCL 150 ms in 20% CO_2_ demonstrating conduction block from atrium to ventricle. **(B)** (i) Effect of acidosis on mean APD_90_ from right atrial pacing. The only significant change demonstrated was at 300 ms. *n* = 5 except PCL 300 ms where *n* = 3; ^*^ = *p* < 0.05. (ii) Effect of acidosis on conduction delay (CD) and 90% action potential duration (APD_90_) from right atrial pacing. As the PCL was decreased in normal pH conditions, there was no significant change in the sum of CD and APD_90_. In acidic conditions, the sum of CD and APD_90_ increased (*n* = 5 except PCL 300 ms where *n* = 3; ^*^p < 0.05).

We then determined the effect of acidosis on mean action potential duration at 90% repolarization (APD_90_) measured from the array of optical traces at each PCL and at each pH. Figure 2A shows that at control pH, APD_90_ was significantly shorter at a PCL of 150 ms than at 300 ms, as expected. During acidosis, no significant difference in APD_90_ was seen across the range of PCLs studied, except at a PCL of 300 ms, at which APD_90_ was significantly shorter in acidosis than at control pH. Figure 2B shows APD_90_ plus conduction delay (CD) at each PCL at control pH and during acidosis. At control pH, increasing PCL had no significant effect on CD+APD_90_, presumably because the increased conduction delay (Figure 1) is compensated by the decrease in APD_90_ (Figure 2A). However, during acidosis the change in APD_90_ fails to compensate for the large increase in conduction delay, so the total time increases. Figure 2B also shows the relation to the threshold for conduction block (PCL+CD) at control pH and during acidosis; at control pH the risk of block is small, as indicated by the time difference between the two relationships at control pH. However, during acidosis the risk of block increases as PCL is decreased, as indicated by the converging lines. In support of the idea that acidosis increases the risk of conduction block as PCL is decreased, intermittent block was observed in all preparations studied at low pH. Taken together, these data indicate that acidosis slowed atrio-ventricular conduction and increased the risk of conduction block. The next series of experiments was undertaken to examine AV nodal conduction in acidic conditions and investigate the pH dependence of these effects.

### The pH dependence of AVN conduction

The use of a HCO_3_/CO_2_ buffer system in the experiments described above, although physiologically relevant, made it difficult to maintain accurate control of pH when using the open chambers necessary for some of the experiments. Consequently, for subsequent experiments we used a HEPES-based buffer system to control pH and define the pH dependence of AV conduction characteristics (see Methods).

### Effect of acidosis on refractory characteristics of the atrium and AVN

Table 2 shows that there was a significant prolongation of the Wenckebach cycle length (WCL) at reduced pH. The table also shows that the AH interval progressively increased after a reduction in pH at PCLs of 300 and 200 ms. Complete heart block occurred in 4 out of 11 hearts at pH 6.3 and a PCL of 300 ms, and in 8 of 11 hearts at pH 6.3 and a PCL of 200 ms. These runs were excluded from the analysis. Thus, consistent with the analysis in the previous section, acidosis slowed conduction and increased the probability of heart block.

**Table 2:** **Effect of pH on AV node conduction characteristics**.

	**pH 7.4**	**pH 6.8**	**pH 6.3**
WCL	198.2 ± 21.5	268.3 ± 29.4^*^	296.0 ± 46.8^*^
AH300	45.8 ± 3.1	54.2 ± 3.5^*^	68.5 ± 7.4^*^
AH200	76.0 ± 26.2	90.9 ± 24.6	-

WCL, Wenckebach cycle length; AH300, AH interval at PCL 300 ms; AH200, AH interval at PCL 200 ms. N = 11 except at pH 6.3 (PCL 300 ms) where n = 7 and at pH 6.3 (PCL 200 ms) where n = 3. (

* *P < 0.05)*.

To investigate the role that changes of refractory period may have in the response to acidosis, the functional and effective refractory periods of the AVN, as well as the effective refractory period of the atrium, were derived at a PCL of 300 ms (as described in Methods). The results are shown in Table 3. Acidosis caused prolongation of each of these refractory periods; the relative change in AVN FRP, AVN ERP, and Atrial ERP at pH 6.8 was to 114 ± 5, 119 ± 3, and 130 ± 7% respectively. At pH 6.3 the change from control conditions was to 131 ± 4, 143 ± 6 and 165 ± 9% respectively. The mean data suggest that the refractory period of the AVN reached a plateau at pH 6.8 and that decreasing pH further to 6.3 had no further effect of refractory period. However, at pH 6.3, in 3 out of 8 preparations, shortening PCL from 300 ms to only 295 ms resulted in immediate conduction block so that it was not possible to derive FRP or ERP. These preparations were therefore excluded from the analysis at pH 6.3. In samples with persistent conduction through to pH 6.3, the FRP and ERP prolonged further at pH 6.3 compared to pH 6.8. This prolongation of the refractory period of the AVN is consistent with an increased probability of heart block during acidosis.

**Table 3:** **Effect of pH on atrial and AV nodal refractory characteristics**.

	**pH 7.4**	**pH 6.8**	**pH 6.3**
AVN FRP	205.1±21.7	237.6±18.4^*^	220.7±27.4^*^
AVN ERP	176.8±22.0	210.7±16.9^*^	209.3±30.6^*^
Atrial ERP	120±22.3	153.3±19.3^*^	187.5±27.7^*^

AVNFRP, AV nodal functional refractory period; ERP, effective refractory period. n = 11 except at pH 6.3 where n = 3 (

* *P < 0.05)*.

### Effect of acidosis on optically derived activation times

**Figure 3:**
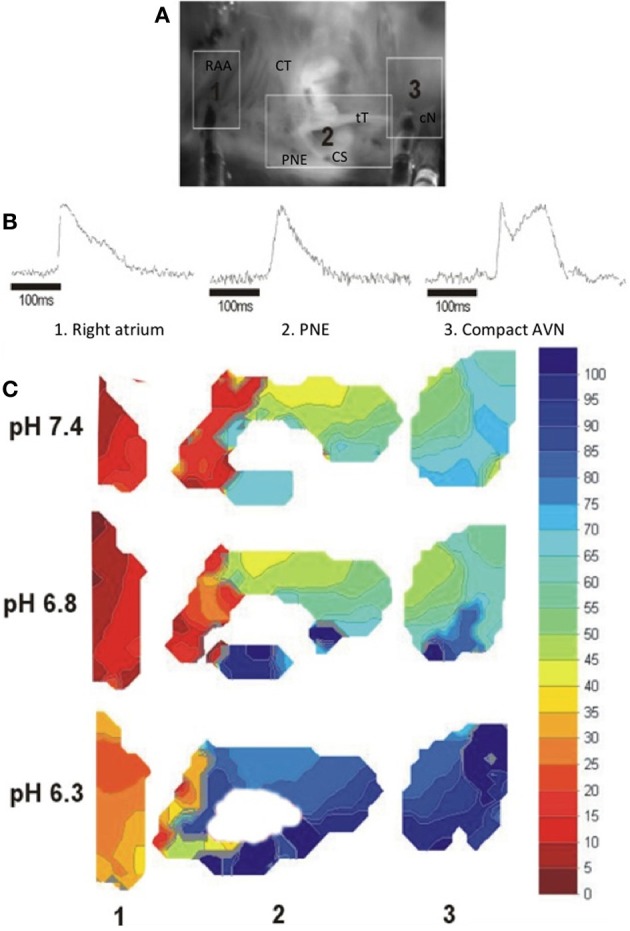
**(A)** CCD image of an isolated right atrial preparation, the areas shown correspond to: Region 1: right atrium in region of the pectinate muscles of the right atrial appendage (RAA) to the crista terminalis (CT); Region 2: Tendon of Todaro (tT) and posterior nodal extension (PNE) surrounding the mouth of the coronary sinus (CS); Region 3: Compact AVN (cN) and His bundle region at the apex of the triangle of Koch. **(B)** Typical optical action potentials from the isolated AV node preparation at normal pH in the regions shown in **(A)**. The multi-component signal at region 3 represents initial compact nodal activation followed by ventricular activation in this complex multi-layered region (Efimov and Mazgalev, 1998). **(C)** Isochronal maps of activation showing conduction delay with reducing pH. Local activation times were measured relative to the onset of the atrial electrogram. Isochronal crowding occurred at the junction of the RA and the posterior nodal extension, with marked delay along the PNE region corresponding anatomically to the slow pathway (Choi and Salama, 1998).

The effect of acidosis on conduction through the AVN was monitored optically, as described in Methods, to determine directly the site showing the largest delay. The upper panel of Figure 3 shows a CCD image of an isolated right atrial preparation, with the areas representing atrium proximal to the node (region 1), the posterior nodal extension leading to the compact node (region 2) and the exit from the compact node to the bundle of His (region 3) highlighted. Representative action potentials measured optically from these 3 regions are shown in Figure 3B. Isochronal activation maps based on the optically derived action potentials at pH 7.4, 6.8, and 6.3 are shown in Figure 3C (with a millisecond time-scale) relative to the onset of the atrial action potential. In region 2 (proximal to compact AVN) there is an area of conduction delay between the proximal and compact AVN, which was observed at every pH, that increased with reducing pH. It is also interesting that in region 2, which corresponds to the area surrounding the coronary sinus, there is an increase in conduction time along the base of the coronary sinus compared to the superior aspect of this region, an area anatomically and functionally considered the “slow pathway” of the AVN. The magnitude of conduction delay occurring at pH 6.8 in region 2 is much greater in the area corresponding to the slow pathway than that superior to the coronary sinus. Region 3 (exit from the compact node to the His bundle) shows relatively little delay, and relatively little effect of acidosis. These data suggest, therefore, that conduction through the slow pathway of the AVN is particularly sensitive to acidosis and that the slow pathway may, therefore, play an important role in the slowed AVN conduction observed in the intact heart during acidosis.

**Figure 4:**
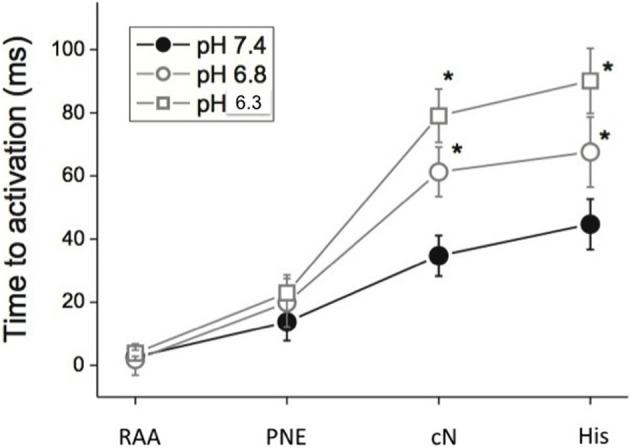
**Time to activation (Mean ± s.e.m. ms) relative to the atrial electrogram at pH 7.4, 6.8, and 6.3 and PCL 300 ms**. (RAA, right atrial appendage; PNE, posterior nodal extension; cN, compact AVN; His, His bundle region). *n* = 3 at each pH.

Figure 4 shows the effect of pH on mean activation time of the optically derived action potential (T_act_) during sinus rhythm. The data obtained during this spontaneous sinus activation show that lowering pH from 7.4 to 6.8, and 6.3 progressively and markedly increased the time to activation of the compact node and His bundle when measured relative to the activation of the posterior nodal extension (ANOVA *p* < 0.001), with little increase in the time to activation from RAA to the posterior nodal extension, compatible with the observation that acidosis had little effect of atrial conduction (above). The slope of the line for each segment shows that, at pH 7.4, there was a small delay between the atrial and PNE regions, and between the compact AVN and His bundle. However, acidosis had little effect on conduction through these regions (the slope remains relatively constant). In contrast, at pH 6.3 there was little delay between the right atrium and the entry to the posterior nodal extension (PNE), but conduction through the PNE region to the compact node was markedly slowed by acidosis. A similar pattern, but less pronounced, was observed during right atrial pacing at a PCL of 250 ms, although there was no conduction of the atrial impulse in 2 of 3 samples at pH 6.3, precluding statistical analysis.

## Discussion

### Effects of acidosis on the AV junction

The present study provides the first direct evidence that acidosis significantly alters the function of the AVN, slowing conduction and prolonging the refractory period, manifested in the whole heart as slowed AV conduction and, at short PCL and low pH, complete AV conduction block.

The observation that acidosis increases the time from atrial stimulation to first epicardial activation, with no significant effect on atrial or ventricular conduction suggests that acidosis inhibits action potential conduction through the AVN. This is consistent with previous work on perfused rat hearts, showing acidosis-induced prolongation of the P-R interval with little change in the QRS complex (Aberra et al., 2001) and was confirmed by our measurements from an isolated right atrial preparation in which the delay was measured directly via activation times. Interestingly, optical measurements suggest that the effect of acidosis on conduction through the AV junction is most marked in the region of the posterior nodal extension, which is known to have clinically relevant slower conduction properties (“slow pathway”) than the superior inputs to the compact node (also referred to as the “fast pathway”) (Mendez and Moe, 1966; Mazgalev and Tchou, 2000; Nikolski and Efimov, 2001; Nikolski et al., 2003; Hucker et al., 2005). This delay was accompanied by an increase in the functional and effective refractory periods of the AVN. In some preparations, these changes were accompanied by and, it appears likely, resulted in, block of AV conduction. The changes in the electrical characteristics of the AVN were more marked at lower pH, and were rate-dependent, becoming more marked as PCL decreased. The possible mechanisms and consequences of these changes are considered further below.

### Possible mechanisms of the effect of acidosis on AVN function

In the present study, acidosis was mimicked by exposing the preparation to an acidic extracellular solution, which in turn decreases intracellular pH. We have previously shown that changing the extracellular pH of isolated AVN myocytes from 7.4 to 6.3 results in a rapid and sustained decrease of intracellular pH, from pH 7.24 to 6.45 (Cheng et al., 2009). Thus, the effects observed here could result from changes of intracellular and/or extracellular pH. Many previous studies have investigated the effect of acidosis on the electrical activity of atrial and ventricular myocytes. Broadly speaking there are two possible sites of action to explain the slowed conduction in the AV junction observed in the present study, changes in: (i) membrane currents and (ii) cell-to-cell resistance.

In our prior study of isolated AV node cells, reduction of extracellular pH (pH_e_; from 7.4 to 6.8 or 6.3) produced pH-dependent slowing of spontaneous action potential rate and upstroke velocity, and reductions in maximum diastolic potential and action potential amplitude (Cheng et al., 2009). Acidosis has previously been reported to decrease I_Na_,which would be expected to slow conduction (Watson and Gold, 1995), yet Na channels have been reported to be sparse in the compact node region in rabbit (Munk et al., 1996), which appears to be the region most sensitive to acidic conditions in the present study. However, Na channels are present in other regions of the AVN, including the transitional cells of the PNE (Petrecca et al., 1997; Yoo et al., 2006), and acid-induced depression of I_Na_ in these regions may depress conduction velocity. Acidosis (pH_e_ 6.3) decreased L-type Ca current (*I*_Ca,L_) in isolated AV nodal cells, without significant changes in voltage-dependent activation or inactivation (Cheng et al., 2009). Acidosis also reduced the E-4031-sensitive, rapid delayed rectifier current (*I*_Kr_) tail amplitude at −40 mV following command pulses to between −30 and +50 mV, and accelerated tail-current deactivation. In contrast, the time-dependent hyperpolarization-activated current, *I*_f_, was unaffected by acidosis. Background current insensitive to E-4031 and nifedipine was reduced by acidosis. Therefore *I*_f_ is unlikely to be involved in the response of the AVN to acidosis, whilst inhibition of *I*_Ca,L_ and *I*_Kr_ by acidosis may contribute to the observed changes. Calcium channel inhibitors have been shown to change the P-R interval of dogs and rabbits (Giacomini et al., 1985; Nose et al., 1993). This, together with the known importance of *I*_Ca,L_ to AVN AP generation in the rabbit (Zipes et al., 1973; Hancox and Levi, 1994) and the fact that canine AVN block has been shown to occur from Ca^2+^ channel blocker application (Zipes and Fischer, 1974), suggests that inhibition of *I*_Ca,L_ by acidosis may be of particular functional significance.

An increase in inter-cellular resistance could also account for, or contribute to the slowed conduction. Acidosis has previously been shown to increase gap-junction resistance between ventricular myocytes (Noma and Tsuboi, 1987), both directly and as a consequence of the increase in cytoplasmic [Ca] caused by acidosis (White et al., 1990). The posterior nodal extension has been shown to have the lowest Cx43 mRNA and the most abundant HCN4 mRNA expression in keeping with its low conduction velocity and pacemaker activity (Boyett et al., 2006). Cx45 has been shown to be abundant in the posterior nodal extension, compact node and both Cx40 and 45 in the NH region (Boyett et al., 2006). It is therefore notable that Cx45 has been reported to be considerably more pH sensitive than Cx43 (Hermans et al., 1995). Consequently, a reduction in cell-cell coupling via Cx45 may combine with the inhibitory effects of acidosis on ion channels to make the slow pathway particularly sensitive to acidic pH compared to the rest of the cardiac conduction system.

### Relevance of the effects of acidosis on AVN function

The AVN plays a key role in normal cardiac function, but can also play a role in arrhythmogenesis, either exacerbating or protecting the heart from arrhythmias. During normal activity, the AVN acts as a slow AV conduction pathway, allowing the correct relative timing and synchrony of atrial and ventricular contraction; the present study shows that acidosis slows AV conduction, without effect on intra-atrial or intra-ventricular conduction velocity. Under some conditions (low frequency, mild acidosis), this effect is small, although the increased delay may increase the protection provided by the AVN during atrial fibrillation. However, during more severe acidosis (pH 6.3) and at higher stimulation frequencies, the conduction delay became more marked. The question therefore arises as to whether this delay could contribute to acidosis-induced arrhythmogenesis. Figure 2 shows that the likelihood of conduction block through the AVN increases during acidosis as PCL decreases. As conduction delay is antecedent to heart block, it is possible that the increase in conduction delay may be important for the increased risk of arrhythmia in acidosis. The arrhythmogenic effect of these changes may be even greater in conditions of regional acidosis where a gradient would exist with non-acidic tissue, since the threshold for block in normal conditions coincides with the APD_90_ in acidic conditions. AV nodal re-entry was not seen in this study either in control conditions or in acidosis. AVN re-entry depends on the presence of “dual AV nodal physiology,” specifically the presence of two pathways with different conduction and refractory characteristics. During pacing with premature stimuli, when conduction blocks in the so called fast pathway, this facilitates conduction with critical delay in the slow pathway, followed by rapid retrograde conduction up the fast pathway, setting up a re-entrant circuit within the AV node. In acidic conditions, the effect on the AVN appears to be significant conduction slowing and refractory period prolongation involving both fast and slow pathway inputs to the compact node. So despite the optical mapping study showing most marked slowing of conduction along the posterior nodal extension, the degree of acidosis affecting the fast pathway is such that rapid retrograde conduction cannot occur and re-entry is not sustainable.

In summary, the data from this study show that acidosis has marked effects on the conduction properties of the AV junction and that this is likely to be a consequence of effects localized to the AVN, namely slowed nodal conduction and prolonged refractory period. These changes result in increased AV conduction delay and, in extreme conditions, conduction block; these changes may, therefore, contribute to arrhythmogenesis, in particular brady-arrhythmias and heart block in conditions in which the heart is exposed to acidosis, such as myocardial ischemia.

### Conflict of interest statement

Research supported by the British Heart Foundation. The authors declare that the research was conducted in the absence of any commercial or financial relationships that could be construed as a potential conflict of interest
